# Trend in mortality from a recent measles outbreak in Cameroon: a retrospective analysis of 223 measles cases in the Benakuma Health District

**DOI:** 10.11604/pamj.2016.23.135.8630

**Published:** 2016-03-25

**Authors:** Tsi Njim, Kimbong Agyingi, Leopold Ndemnge Aminde, Edwin Fon Atunji

**Affiliations:** 1 Regional Hospital Bamenda, Bamenda, North west region, Cameroon; 2 Health and Human Development (2HD) Research Group, Douala, Cameroon; 3 District Hospital Benakuma, Benakuma, North west region, Cameroon; 4 School of Public Health, Faculty of Medicine & Biomedical Sciences, University of Queensland, Brisbane, Australia; 5 Clinical Research Education, Networking and Consultancy, Douala, Cameroon

**Keywords:** Measles, mortality, sustainable development goals, outbreak response immunisation, Cameroon

## Abstract

**Introduction:**

Measles is a highly contagious viral infection with high mortality in poorly vaccinated regions. We sought to establish the trend in mortality and the factors that favoured the recent measles outbreak that occurred in Benakuma, in the North west region of Cameroon from the 21/06/2015 to 26/09/2015.

**Methods:**

We carried out a retrospective register analysis of 223 measles cases. Time trends were established using the Mann-Kendall test while survival was assessed using the Kaplan-Meier survival analysis and log rank test for comparisons.

**Results:**

We had a case fatality of 8.5% and the mortality decreased significantly (p = 0.01) after the following public health interventions were instituted: community sensitisation and education; outbreak response immunisation and the use of a clinician in controlling the outbreak. Furthermore, the number of cases (p < 0.01) and the duration from onset of illness to consultation at a health facility (p < 0.01) decreased significantly after the institution of the aforementioned interventions. Also, survival during the outbreak was better in females (p = 0.02) and in those treated in health facilities (p < 0.01).

**Conclusion:**

The poor vaccination status in Benakuma coupled with negative cultural beliefs; poor socioeconomic and environmental factors and inadequate public health policies predisposed the region to a measles outbreak and favoured the spread of the virus. Public health policies should be revisited, modified and intensified to scale up vaccination coverage in measles endemic zones in Cameroon to help eliminate the disease and facilitate the overall attainment of the Sustainable Development Goals.

## Introduction

**Figure 1:**
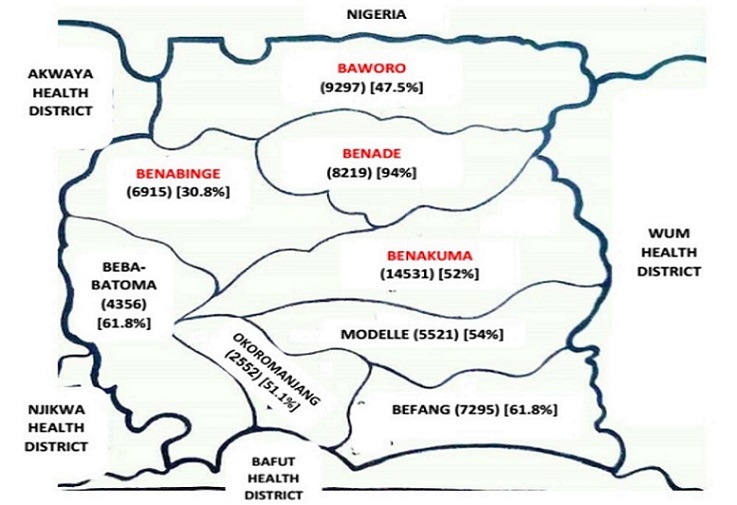
Map of the Benakuma Health district showing Health Areas affected by outbreak (in red), the population of the various health areas (in round brackets) and their vaccination statuses (in square brackets)

Measles is a highly contagious viral disease with considerable morbidity and mortality in children and pregnant women; and is still a leading cause of vaccine-preventable deaths in the world [1, 2]. Measles is endemic in several regions in Cameroon and this could be blamed on the poor vaccination coverage in these regions and the inadequate health policies in place to ensure measles control [3]. Despite the 90% nationwide vaccination coverage that led to a decrease in annual incidence from 41 cases per 100,000 children in 2001-2004 to 2 cases per 100,000 children from 2005-2008 [3, 4]; there are still several regions which have very low vaccination coverage like Misaje (found in the Northwest region of Cameroon) with 78% measles vaccine coverage and Benakuma where vaccination coverage ranges from 30.8% to 94% (Figure 1) [3, 5]. These regions which are highly unvaccinated remain significant pitfalls to measles elimination as over 95% of a population should be immunised in order to achieve this objective [6]. Poor routine vaccination coverage coupled with the absence of Supplementary Immunisation Activities (SIAs) despite Active Disease Surveillance (ADS) has led to frequent outbreaks in measles endemic areas in Cameroon [3]. In this study, we assessed the impact of immunisation and the public health interventions on the trend in mortality during the measles outbreak which occurred in the Benakuma Health District (BHD) in North West, Cameroon.

## Methods

### Study design and setting

A retrospective register analysis was carried out. BHD is 1 of the 19 Health Districts that make up the North West Region of Cameroon. It has a total population of 58677 and shares boundaries with Wum, Bafut, Njikwa and Akwaya Health Districts. BHD is made up of 8 health areas namely: Baworo; Beba-Batoma; Befang; Benabinge; Benade; Benakuma; Modelle; and Okoromanjang health areas. The measles outbreak occurred in 4 of these health areas which have very poor vaccination coverages (Baworo, Benabinge, Benade and Benakuma) (Figure 1). Despite this poor vaccination coverages, there has been no measles outbreak in the region for the past 5 years so the WHO definition of an outbreak is satisfied [7].

### Participants and sampling

Participants included all individuals who presented to any health centre or hospital in the district with suspicion of measles or any individual whom after home investigations by the public health authorities (PHAs) was found to have suffered or died from a suspicion of measles during the period of the outbreak (21st of June 2015 to the 26th of September 2015). A diagnosis of measles was advocated: if an individual had the following symptoms: a generalised febrile rash associated with either conjunctivitis; cough or coryza and in any person in whom a clinician suspected a measles infection.

### Data collection and variables

During the outbreak, the following information was recorded from any child who presented with a suspicion of measles. Socio-demographic data (age and health area), immunisation history (measles vaccination status and year of vaccination), clinical data (date of onset of illness, date of consultation and other symptoms like conjunctivitis, cough and coryza), place of treatment (home or health facility), outcome (death or recovery from illness). Blood samples were collected from 13 of the cases and sent to a reference laboratory; Centre Pasteur du Cameroun (CPC) in the capital city: Yaoundé, for detection of Ig-M antibodies for confirmation of measles. After the outbreak, a report was written by the public health authorities.

### Data management and statistical analysis

Data were cross-checked for errors before entry into a password-protected personal computer and the participants were identified using numbers. Data were analysed using Epi Info version 3.5.4 (CDC, Atlanta, USA) Means and standard deviations were used to summarise continuous variables and proportions for categorical variables. Categorical variables were compared using Fisher's exact tests. Time trends were established using the Mann-Kendall test while survival was assessed using the Kaplan-Meier survival analysis and log rank test for comparisons. Statistical significance was set at p < 0.05.

### Ethical considerations

Approval was obtained from the Ethical committee of the North west Regional Delegation of the Ministry of Public Health.

## Results

A total of 223 measles cases were reviewed. The cases were further divided into the following classification: Laboratory confirmed cases (3.6%), Epi-linked cases (26.5%) and clinically suspected cases (69.9%).

### Sociodemographic characteristics

The mean age of the participants was 3.6 ± 3.0 years. Over half of the cases were from the Benakuma Health Area, the majority were male (55.2%) and about half of the cases had not received any prior measles immunisation (table 1).

**Table 1:** Sociodemographic and clinical characteristics of 223 measles cases during outbreak from 21^st^ of June 2015 to the 26^th^ of September 2015 in the Benakuma Health District, North west, Cameroon

Characteristics	Groups	Number	Proportion (%)
**Sex (N=223)**	Male	123	55.2
	Female	100	44.8
**Age in years (N=223)**	<5	171	77.0
	5 - 16	50	22.5
	>16	1	0.5
**Health area (N=223)**	Benade	35	15.7
	Benakuma	137	61.4
	Baworo	6	2.7
	Benabinge	46	20.2
**Measles vaccination (N=223)**	Yes	32	14.4
	No	106	47.5
	Unknown	85	38.1
**Maculopapular rash (N=223)**	Yes	223	100.0
	No	0	0.0
**Fever (N=223)**	Yes	223	100.0
	No	0	0.0
**Conjunctivitis (N=223)**	Yes	160	71.8
	No	63	28.3
**Cough (N=223)**	Yes	209	93.7
	No	14	6.3
**Coryza (N=223)**	Yes	88	39.5
	No	135	60.5
**Treated at home (N=223)**	Yes	18	8.1
	No	205	91.9
**Outcome (N=223)**	Deceased	19	8.5
	Alive	204	91.5

### Primary case

The index case was an 8 year old male who had a sudden onset febrile rash, cough, coryza and conjunctivitis on the 21/06/2015. He was managed at home in the Benakuma health area traditionally with palm wine and herbs. The illness worsened with the onset of severe diarrhea and he died at home five days later (on the 26/06/2015). His other three siblings later on developed signs and symptoms suggestive of measles, two of whom died at home while the third was carried to the Benakuma District Hospital by on field investigators where she was managed.

### Secondary cases

Most of the secondary cases from the Benade, Baworo and Benabinge health areas all had contact with the primary case and other secondary cases in the Benakuma health area during solidarity visits. All the patients managed in the health facilities were placed on supportive therapy and Vitamin A.

### Clinical characteristics

**Figure 2:**
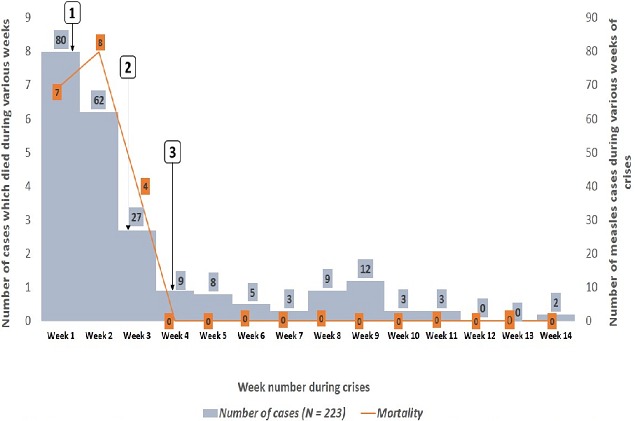
Weekly interventions during the outbreak plotted against the number of cases and the mortality

All the participants had a generalised maculopapular rash and fever with the most common other symptom being cough in 93.7% of the study population (Table 1). Most of the participants presented during the first week of the outbreak (35.9%). The trend in incidence decreased steadily over the weeks (p<0.01) (Figure 2). The mean duration from onset of illness to presentation at a health facility was 4.5 ± 3.3 days. A decreasing trend in this mean duration was observed as the outbreak progressed (p < 0.01) (Table 2). During this outbreak, 8.1% (n=18) of the 223 cases were treated at home; and 8.5% (n=19) of the cases died (Table 1). All the cases managed at home died (p<0.01). The trend in overall mortality decreased steadily over the weeks of the outbreak (p = 0.01). Survival was better in females with a 3 week survival probability of 95.0% and males 81.0% (log-rank: 5.1, p = 0.02) (Table 3), in patients managed in health facilities (log-rank: 225.3, p < 0.01) (Table 3), and in children who had been vaccinated at least once against measles (Table 3) (log-rank: 2.9; p = 0.09).

**Table 2:** Weekly mean duration from onset of illness to presentation at a health facility for 223 measles cases during outbreak from 21st of June 2015 to the 26th of September 2015 in Benakuma Health District, North West, Cameroon

Weeks	1	2	3	4	5	6	7	8	9	10	11	12	13	14
Mean*	7.2	3.8	2.2	3.1	2.6	1.6	2.0	3.7	1.6	2.0	1.0	NA	NA	1.0
SD	3.2	1.8	1.6	2.5	1.8	0.5	0.0	3.9	0.8	1.0	0.0	NA	NA	0.0
N	80	62	27	9	8	5	3	9	12	3	3	0	0	2

* Mean duration from onset of illness to presentation at the hospital for consultation

SD: Standard deviation; N: Number of cases; NA: Not applicable.

**Table 3:** Survival probability of patients according to gender, locum of case management (home vs. health facility) and vaccination status

			Survival probability				
		Week 1	Week 2	Week 3	Week 4	Log-rank	p value
**Prior vaccination status**	**Yes**	1.00	1.00	1.00	1.00	2.9	0.09
	**No/status unknown**	0.97	0.90	0.85	0.85		
**Managed in health facility**	**Yes**	1.00	1.00	1.00	1.00	225.3	< 0.01
	**No**	0.60	0.21	0.00	0.00		
**Sex**	**Males**	0.97	0.87	0.81	0.81	5.1	0.02
	**Females**	0.97	0.97	0.95	0.95		

### Public health interventions

Public health authorities (PHAs) were informed of an outbreak on 26/06/2015, 5 days after the onset of the outbreak as this was the time of arrival of the first cases to health facilities in the district. The next day, health care providers (HCP) were sent to the field for 8 days to: search for new cases or old unrecorded cases; establish the limits of the zones affected; sensitize the population on the signs and symptoms of measles and what to do when a child had suggestive symptoms; sensitize HCPs on surveillance of measles (performing community contact tracing and educating contacts on symptoms and the need to present at health facilities early); brief staff on harmonized measles case management; conduct verbal autopsy for the reported deaths related to suspected measles; review of consultation registers in the health units and health education of the community especially to deter rumours that if a child has measles and is injected the child will die. A medical officer was sent to the health district on 15/07/2015 to ensure better clinical management of the cases. Finally, outbreak response immunisation was commenced on the 16/07/2015 and the four areas involved (Figure 1) were covered. The outbreak lasted from the 21/06/2015 to 26/05/2015 (14 weeks) with 223 cases reported (Figure 2).

## Discussion

Measles has a high secondary infection rate and a high mortality especially in children [1]. The case fatality in our study was quite high (8.5%) when compared to those obtained during other measles outbreaks in Cameroon: 2.2% in the North of the country in 2010; 0% in Nylon, Douala in 2011 and 0% in Misaje in 2015 [3, 8]. The frequency and severity (case fatalities) of measles outbreak is known to be highly responsive to vaccination activities [4, 9, 10]. Routine vaccination in the Benakuma health district is however very poor. The general poor vaccination status in this region was demonstrated in this study as only 14.4% of the cases were confirmed to have received at least 1 measles vaccine (Table 1). The vaccination coverage for 2015 showed that 7 of the 8 health areas in this district could barely vaccinate half of their target population (Figure 1). This poor vaccination coverage could be blamed specifically on the inaccessibility of most regions to health facilities and insufficient staff in the health facilities. The health facility in Baworo has just 1 HCP and is situated 15km from Banum II the main affected village in this health area. Accessibility is by trekking for several kilometres covering hills and crossing rivers, hence even the HCP finds it difficult to cover significant populations during outreach vaccination campaigns. The Benabinge Health Area with population of 7295 has no staff and no health unit. Vaccination is done through outreach activities and 95% of the health area is only accessible by trekking over steep hills and large rivers. The affected villages of Ekuru and Akeremise in this health area are situated over 20km from the nearest health facility (Benakuma District Hospital). There is no refrigerator so vaccines are taken from Benakuma and used for outreach under doubtful storage conditions. The Benade Health Area with just 1 staff went operational in 2013, hitherto vaccination was done by outreach community volunteers. The affected village of Benahundu in this health area is 8km from the Health centre with no outreach post. The Benakuma Health Area is very vast with a population of 14,531 inhabitants and just 3 nurses. Outreach in the affected zone of Bosung in this health area is once a month by community volunteers who trek over hills and rivers to arrive at village settlements. Also, the lack of SIA in most regions in the country is worrisome. Before this outbreak, there had been no SIA for over 10 years. This poor routine vaccination coupled with the fact that most children do not receive a second dose of the measles vaccine as it is not provided in the Extended Program of Immunisation in Cameroon shows that most populations remain poorly vaccinated and unimmunised; hence ensuring that measles remains endemic and increasing the likelihood of outbreaks. This outbreak like others in the country could therefore be blamed on poor vaccination coverage [3, 4, 8] and reaffirms the assertion that measles remains an endemic disease in the country with a low possibility of elimination or eradication as over 95% of the population of a community have to be vaccinated to ensure immunisation [6].

This is in great contrast to developed countries with high vaccination coverage where outbreaks are rare and could only be blamed on importation from measles endemic zones [2, 11, 12]. Also, the poor socioeconomic and environmental factors and the negative cultural beliefs in this region seemed to favour the spread of the virus during the outbreak. Solidarity visits which most of the time involved young children were rampant in the early weeks of the outbreak. Furthermore, the inhabitants have no toilets and personal and environmental hygiene needs a lot of improvement as sleeping units are overcrowded and poorly ventilated. The above situation greatly favoured the rapid spread of the epidemic. Persistent poor vaccination coverage in Cameroon coupled with inadequate public health policies [3] could pose a significant pitfall towards the attainment of the Sustainable Development Goals (SDGs) in the post 2015 era; especially Goal 3 which has as one of its targets ending the epidemics of AIDS, tuberculosis, malaria, and neglected tropical diseases and combat hepatitis, water-borne diseases, and other communicable diseases by 2030 [13]. The role of the PHAs during this outbreak was pivotal. The case fatalities and the number of cases decreased considerably as soon as the public health interventions were put in place (Figure 2) and these trends were both significant. In the first week of the outbreak, PHAs were mobilised and sent to the field. They sensitised and educated the population on the signs and symptoms of the disease and informed the community on the risks of solidarity visits during the outbreak. This led to an immediate drop in the number of cases in week 2, a trend which continued throughout the outbreak. Also, the PHAs emphasised the need to manage children with features suggestive of measles at health facilities and greatly dispelled the negative rumours in the society that if a child with measles was given intravenous infusions the child would die. These actions carried out in week 1 actively decreased the case fatalities as shown in Figure 1; especially as this demonstrated that children who were treated at home had a lower survival probability (Table 3). The actions also helped to decrease the time from onset of illness to presentation at a hospital which showed a downward statistically significant trend over the weeks of the outbreak; a factor which indirectly helped decrease the mortality. Furthermore, the role of ORI has been shown to be crucial in managing measles outbreaks and in reducing measles related mortality [4]. This assertion was also confirmed in this study. This immunisation exercise was commenced in the third week of the outbreak in all the 4 health areas affected. After the exercise started, the number of case fatalities decreased considerably (Figure 2). Finally, this population did not have a medical officer before the outbreak. A medical officer was sent to handle the outbreak in the third week. His presence seemed to help decrease the number of cases over the weeks and the case fatalities. Other authors have also supported an important role by clinicians in resource-limited settings in the control measles outbreaks [3]. This study was limited by the following potential pitfalls: being a retrospective study, there was always a possibility that some of the records may not have been correctly filled. Also the study may have underestimated the extent of the measles outbreak as not all individuals who were sick presented at the health facilities. The HCPs sent to the communities to investigate potential measles victims may also have missed some cases.

## Conclusion

Measles outbreaks in regions with poor vaccination coverage could be associated with a high mortality especially when the spread is favoured by negative cultural, socioeconomic and environmental factors. However, significant interventions like intense community sensitisation and education; ORI and the input of HCPs with adequate knowledge on measles control seem to be pivotal in the control of outbreaks and reducing the associated mortality. Also, public health policies especially on routine vaccinations and SIAs need to be modified and or intensified to upgrade measles endemic and poorly vaccinated regions to fully immunised populations. Such modifications could be promising towards overall attainment of the SDGs in Cameroon.

### What is known about this topic

- Measles is a high contagious viral infection with a high mortality amongst children and pregnant women
- Measles outbreaks are rare in regions where adequate routine vaccination is coupled with supplementary immunisation activities and active disease surveillance
- Measles outbreaks are highly responsive to vaccination campaigns

### What this study adds

- Some regions in Cameroon remain endemic to measles due to poor vaccination statuses
- The severity of measles outbreaks can be favoured by negative cultural beliefs
- The role of health care providers in dispelling myths surrounding the disease during outbreaks cannot be underestimated
